# Trek1 contributes to maintaining nasal epithelial barrier integrity

**DOI:** 10.1038/srep09191

**Published:** 2015-03-17

**Authors:** Jing Jiang, Jiang-Qi Liu, Jing Li, Meng Li, Hong-Bin Chen, Hao Yan, Li-Hua Mo, Shu-Qi Qiu, Zhi-Gang Liu, Ping-Chang Yang

**Affiliations:** 1 Shenzhen Key Laboratory of Allergy & Immunology, ENT Institute of Shenzhen University, 518060 Shenzhen, China; 2 ENT Hospital, Longgang Central Hospital, 518116 Shenzhen, China; 3 Brain Body Institute, McMaster University, Hamilton, ON L8N 4A6 Canada

**Keywords:** Immunological disorders, Cell adhesion

## Abstract

Epithelial barrier integrity is critical to maintain the homeostasis in the body. The regulatory mechanism of the epithelial barrier function has not been fully understood. This study aims to elucidate the role of the TWIK-related potassium channel-1 (Trek1) in the regulation of the epithelial barrier function of the nasal mucosa. In this study, the levels of Trek1 were assessed by real time RT-PCR and Western blotting. The epithelial barrier function of the rat nasal epithelia was evaluated by the Ussing chamber system. The results showed that Trek1 was detected in the human and rat nasal epithelia, which were significantly lower in patients and rats with allergic rhinitis than that in healthy controls. Exposure to the signature T helper 2 cytokine, interleukin (IL)-4, markedly suppressed the expression of Trek1 in the nasal mucosa via up regulating the expression of the histone deacetylase (HDAC)1. The IL-4-induced rat nasal epithelial barrier dysfunction could be blocked by HDAC1 inhibitor (Trichostatin A), or sodium butyrate, or administration of *Clostridium Butyricum*. We conclude that Trek1 is critical to maintain the nasal epithelial barrier function.

**Electronic supplementary material:**

The online version of this article (doi:10.1038/srep09191) contains supplementary material, which is available to authorized users.

## Introduction

The physical components of the epithelial barrier consist of the epithelial cell bodies and the tight junctions. Only water and small molecules can pass through the epithelial barrier to enter the deep tissue under healthy conditions. The epithelial barrier may be disrupted in unusual circumstances; such as psychological stress^1,2^, allergic responses^3^, inflammation^4^ and infections^5^. The mechanisms of epithelial barrier dysfunction have been investigated extensively. However, the regulatory factors of the epithelial barrier integrity have not been fully elucidated yet.

The hyperpermeability is one of the major features of the epithelial barrier dysfunction, in which macromolecular antigens or noxious substances may pass through the barrier to reach the subepithelial region to contact immune cells to initiate unwanted immune responses. It is suggested that extrinsic molecules may pass through the epithelial barrier via the paracellular pathway or the intracellular pathway^6^. In the former case, the paracellular space may be enlarged by losing tight junction associated proteins^2,7^, or increasing in the expression of some tight junction associating proteins, such as claudin 2^8^. In the latter case, the endocytic molecules are not properly decomposed in the epithelial cells, such as deficiency of ubiquitin A20^9^, resulting in the macromolecular proteins or peptides with functional antigenicity to be transported across the epithelial barrier to reach the subepithelial region. The mechanism of the epithelial barrier dysfunction has not been fully understood yet.

Histone deacetylases (HDAC) are a group of enzymes that remove an acetyl group from lysine amino acid on a histone, which allows the DNA to be wrapped by the histone to prevent the gene transcription. HDAC activities are involved in multiple cell activities, including inflammation^10^, cancer cell growth^11^ and allergic disorders^12^. HDAC1 can bind to the Fas promoter in T cells and butyrate inhibits the HDAC1 activity to induce Fas promoter hyperacetylation and Fas upregulation in T cells^13^. Epithelial barrier dysfunction is associated with the pathogenesis of allergic diseases and inflammation; whether HDAC regulates the epithelial barrier function is to be investigated.

The TWIK-related potassium channel-1 (Trek1) is the K2P channel (Two Pore Domain Potassium Channels), an important member of the family. Earlier studies of the expression and function of TREK 1 are more concentrated in neural systems^14,15^, recent studies showed that TREK 1 expression in endothelial cells mediated vasodilation and local regulation^16^.

Although its original function is for potassium ion transportation, recent reports have revealed that Trek1 plays a critical role in the maintenance of the endothelial barrier integrity in the brain blood barrier^15^ and HDAC1 can suppress Trek1^15^. Whether Trek1 and HDAC1 are involved in the airway epithelial barrier integrity has not been investigated. Based on the above information, we hypothesize that Trek1 plays a role in the maintenance of the epithelial barrier function. The results of the present study showed that the human and rat nasal epithelia expressed Trek1, which was suppressed by allergic responses via modulating the expression of HDAC1.

## Results

### Trek1 is suppressed in the nasal epithelia of patients with allergic rhinitis (AR)

**Figure 1:**
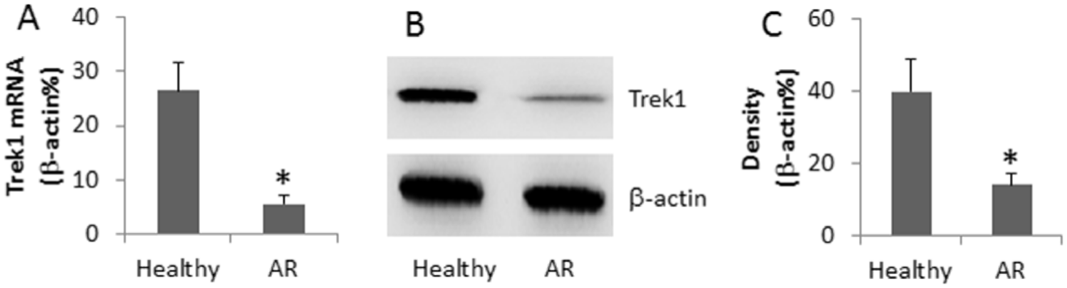
Assessment of Trek1 in the nasal epithelia. Nasal epithelia were collected from 30 healthy subjects and 30 AR patients. The extracts of RNA and protein were analyzed by qRT-PCR and Western blotting. (A), the bars indicate the mRNA levels of Trek1. (B), the immune blots indicate the protein levels of Trek1. (C), the bars indicate the integrated density of the immune blots of (B). The data are presented as mean ± SD. *, p < 0.01, compared with the healthy group. Specimens from individual patients were analyzed separately. The data are a representative of 30 independent experiments.

Recent reports indicate that Trek1 is involved in maintaining barrier function^15^. Epithelial barrier dysfunction is one of the factors in the pathogenesis of AR. Thus, we assessed the levels of Trek1 in the nasal mucosa. Nasal epithelial specimens were collected from patients with AR and healthy subjects and assessed by qRT-PCR and Western blotting. The results showed that Trek1 was detected in the normal nasal epithelia, which was much less in the specimens from AR patients (Fig. 1). The results implicate that allergic response suppresses the expression in the nasal epithelia.

### Allergic responses inhibit Trek1 to compromise nasal epithelial barrier function

**Figure 2:**
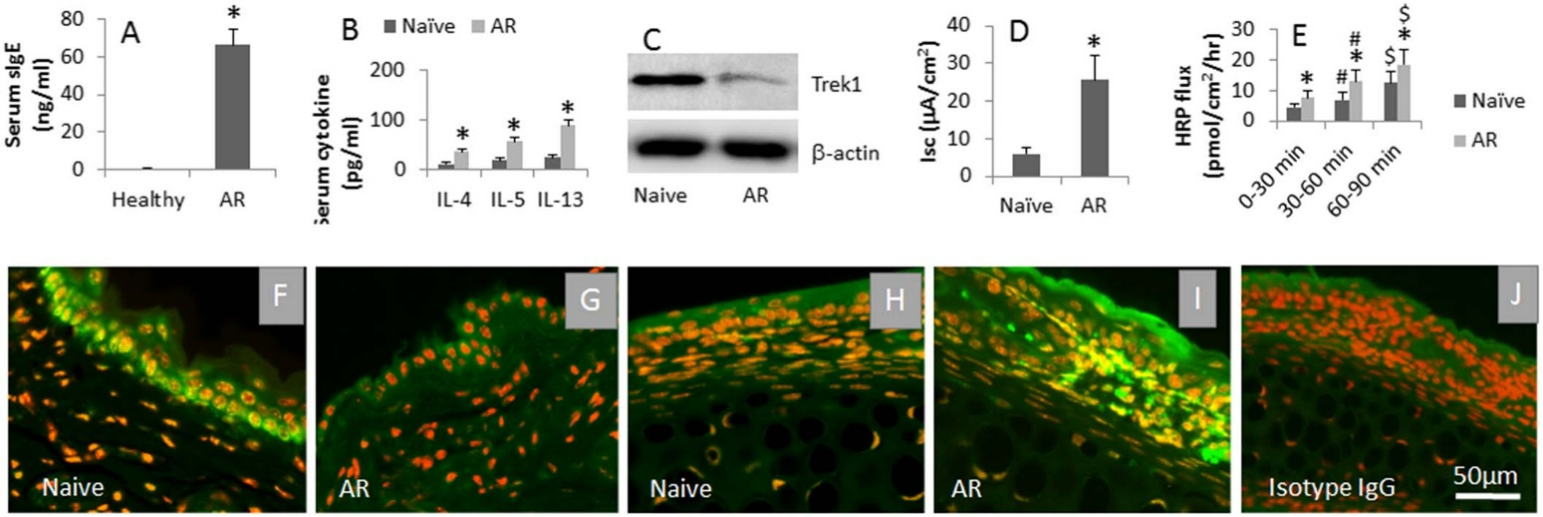
Allergic response suppresses Trek1 in nasal epithelia. Rats were sensitized to OVA. A–B, ELISA data; the bars indicate the serum levels of specific IgE (A) and Th2 cytokines (B). C, the immune blots indicate the protein levels of Trek1 in nasal epithelia (by Western blotting). (D–E), (data of Ussing chamber); the bars indicate that the Isc of the nasal epithelia (D) and HRP flux (E). F–I, the representative confocal images show the positive staining (in green) of Trek1 (F, G) and HDAC1 (H, I). (J), isotype control. AR: Allergic rhinitis. The data are presented as mean ± SD. *, p < 0.01, compared with naïve group. #, p < 0.05, compared with 0–30 min. $, p < 0.05, compared with 30–60 min. Each group consists of 6 rats. Samples from individual rats were processed separately. The data are a representative of 6 independent experiments.

Allergic rhinitis is an IgE-mediated disorder, in which antigen specific IgE bounds on the surface of mast cells to make mast cells sensitive. Re-exposure to specific antigens induces the mast cells to release inflammatory mediators and evoke allergic inflammation^17^. It is documented that the epithelial barrier dysfunction is associated with the pathogenesis of allergic diseases^18^. Whether allergic responses affect Trek1 expression has not been investigated. To this end, we created a rat nasal allergy model. The allergic rats showed that, after specific antigen challenge, nasal itch (6/6; 100%), increases in nasal secretion (6/6; 100%) and high levels of serum specific IgE (Fig. 2A) and Th2 cytokines (Fig. 2B). The levels of Trek1 in the allergic rat nasal mucosa were significantly lower than the control rats (Fig. 2C). The nasal epithelial barrier function was assessed by Ussing chamber technique. As compared to the control group, the Isc (Fig. 2D) and permeability (Fig. 2E) to the specific antigens were markedly higher in the allergic group. The results were further supported by the data of immunohistochemistry (Fig. 2F-J). The results indicate that the expression of Trek1 is suppressed in the nasal mucosa with allergic disorders, which is in parallel to the nasal epithelial barrier dysfunction.

### Allergic mediators increases HDAC1, the HDAC1 suppresses Trek1 expression in the nasal mucosa

**Figure 3:**
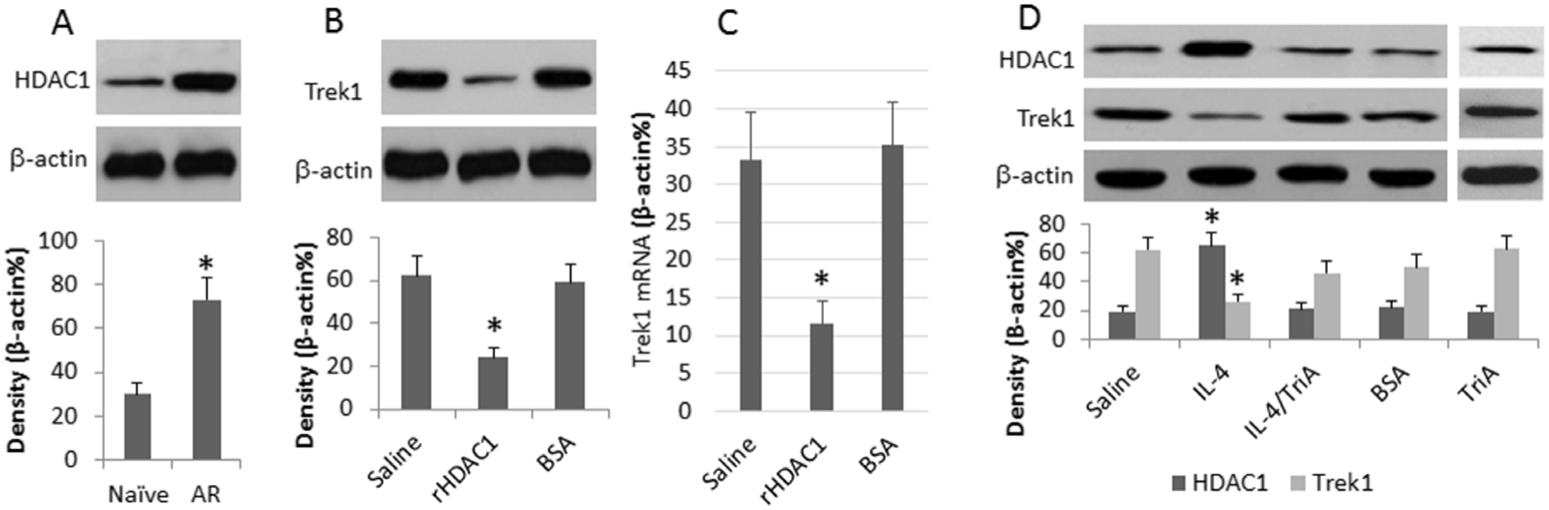
Th2 cytokine, IL-4, suppresses Trek1 via HDAC1. (A), nasal epithelial specimens from naïve rats and AR rats were analyzed. The immune blots indicate the HDAC1 levels. (B), naïve rats were treated with a nasal drop of HDAC1 (1 μg/ml saline) daily for 7 days. The immune blots indicate the Trek1 levels in the nasal epithelia. (C), the bars indicate the mRNA levels of Trek1 in the rat nasal epithelia. (D), rats were treated as denoted on the X axis. TriA, Histone deacetylase inhibitor (Trichostatin A, 1 μM in the nasal drop). The immune blots indicate the levels of HDAC1 and Trek1. The data are presented as mean ± SD. *, p < 0.01, compared with naïve group (A), or saline group (B, C, D). The data are a representative of 6 independent experiments.

HDAC1 is involved in a number of inflammatory processes^19^ including allergic disorders^20^. We inferred that the suppression of Trek1 in the nasal mucosa might be associated with HDAC1 activities. To test the hypothesis, we assessed the levels of HDAC1 in the nasal mucosal samples of Fig. 2. The results showed that the levels of HDAC1 were higher in the nasal mucosa of allergic rats than the naive control rats (Fig. 3A), which implicate that HDAC1 may inhibit the expression of Trek1 in nasal epithelial cells. To test the inference, we treated naïve rats with a nasal drop of recombinant HDAC1 daily for 7 days. The nasal mucosa was excised upon the sacrifice and analyzed by Western blotting. The results showed that the administration of rHDAC1 markedly suppressed the expression of Trek1 in the nasal mucosa as compared to the rats treated with BSA (Fig. 3B, 3C).

On the other hand, we treated naïve rats with nasal drops of the Th2 signature cytokine, IL-4, daily for 7 days. The treatment significantly increased the expression of HDAC1 in the nasal mucosa as well as a marked suppression of the expression of Trek1 (Fig. 3D). To elucidate if the suppression of Trek1 was mediated by HDAC1, in separate experiments, an inhibitor of HDAC1 was used together with IL-4 to treat rats in the same procedures above. The suppression of Trek1 in the nasal mucosa was abolished (Fig. 3D). Treating normal rats with the inhibitor of HDAC1 alone did not suppress the HDAC1 below its normal levels in the nasal mucosa, nor affected the expression of Trek1 (Fig. 3D).

### Inhibiting HDAC1 ameliorates allergic response-induced nasal epithelial barrier dysfunction

**Figure 4:**
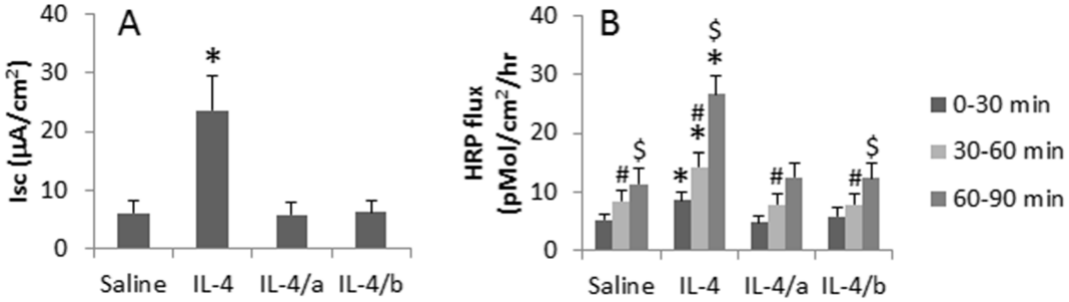
IL-4 compromises nasal epithelia barrier integrity. Naïve rats were treated with a nasal drop containing IL-4 (100 ng/ml) daily for 7 days. The nasal mucosa was assessed with a mini Ussing chamber system. (A), the bars indicate the Isc levels. (B), the bars indicate the HRP levels passing across the nasal epithelial layers./a, a HDAC1 inhibitor (Trichostatin A, 1 μM in the nasal drop) was added to the nasal drop./b, the rats were gavage-fed with *C. butyricum* (10^9^ bugs/rat/day). The data are presented as mean ± SD. *, p < 0.01, compared with saline group. #, p < 0.01, compared with 0–30 min. $, p < 0.01, compared with 30–60 min. The data are a representative of 3 independent experiments.

To corroborate the above results, we treated rats with a nasal drop containing Th2 mediators with or without the HDAC1 inhibitor daily for 7 days. After sacrifice, the nasal epithelial barrier function of the rats was assessed by an Ussing chamber system. The results showed that the administration of HDAC1 inhibitors effectively prevented the Th2 cytokine-induced epithelial barrier dysfunction (Fig. 4). The probiotic *Clostridium butyricum* produces butyrate; the latter is a kind of HDAC inhibitor. We inferred that administration of *C. butyricum* might reconcile the IL-4-induced epithelial barrier dysfunction. To test the hypothesis, we treated naïve mice with a nasal drop of IL-4 in combination of gavage-feeding *C. butyricum* daily for 7 days. As shown by the Ussing chamber assessment, the IL-4-induced epithelial barrier dysfunction was abolished by the gavage-feeding with *C. butyricum* (Fig. 4).

## Discussion

Since the epithelial barrier function is critical to maintain the homeostasis of the body, to investigate the regulatory factors of the epithelial barrier function is of significance. The present study has revealed a novel molecule, Trek1, which plays a critical role in the nasal epithelial barrier integrity. The results showed that both the human and rat nasal epithelia expressed Trek1. Allergic responses suppressed the expression of Trek1 in the nasal epithelia via up regulating HDAC1. Accordingly, blocking HDAC1 prevented the allergic response-suppressed Trek1 in the nasal epithelia and the allergic response-induced nasal epithelial barrier dysfunction. The results suggest that HDAC1 may be a potential target for the treatment of nasal allergy.

Trek1 is a potassium channel with a primary function of the potassium ion transportation. However, more functions have been identified on this protein. Roan et al indicate that TREK-1 regulates stretch-induced detachment of alveolar epithelial cells^21^. Brohawn et al suggest that mechanosensitivity is mediated directly by the lipid membrane in TRAAK and TREK1 K^+^ channels^22^. The ion channel also plays a role in determining appropriate maintenance of uterine quiescence during pregnancy^23^. Our data demonstrate that Trek1 is required in maintaining the nasal epithelial barrier function; the data are in line with a recent report that Trek1 plays a critical role in the barrier function of the brain blood barrier^15^, in which the authors demonstrate that the deficiency of Trek1 results in a serious dysfunction of the brain blood barrier.

The activation of HDAC reduces the gene transcription activities. The present data are in line with this notion by showing that the increase in HDAC1 is involved in the allergic response-induced Trek1 suppression in the nasal epithelia. Others report that treatment with HDAC inhibitors reduces peroxisome proliferator-activated receptor γ levels in differentiated adipocytes^24^; Yao et al indicate that death domain-associated protein 6 represses IL-6 expression via activating HDAC1^25^. Activation of HDAC1 is involved in the pathogenesis of lung inflammation^26^; whether the nasal allergic inflammation is associated with the activities of HDAC1 is to be further investigated. HDAC1 also modulates the activities of other proteins, such as decreasing the transcriptional activity of the deacetylated protein with p53^27^. Inhibition of histone deacetylases can induce apoptosis and to sensitize cells to chemotherapy or radiotherapy^28^.

IL-4 is a signature cytokine of Th2 response. Previous studies indicate that IL-4 directly compromises the epithelial barrier function [Wise SK, 2014 23/id]. Our results are in line with the report by showing that exposure to IL-4 increases the Isc and the permeability to HRP in the nasal epithelial layer. Berin et al also report that IL-4 compromises the intestinal epithelial barrier function^29^. The present data have provided further insight of the mechanism by which the allergic mediator IL-4 affects the nasal epithelial barrier function by increasing the expression of HDAC1 and suppressing the expression of Trek1 in the nasal epithelia. Of course, Th2 cytokines include several other cytokines, such as IL-5 and IL-13; whether these Th2 cytokines also affect the expression of Trek1 needs to be further investigated.

The anti-inflammatory properties of probiotics are recognized; but the underlying mechanism has not been elucidated yet. Schwarzer et al report that the neonatal colonization of germfree mice with *Bifidobacterium longum* can prevent allergic sensitization to major birch pollen allergen Bet v 1 Bet v 1^30^. Although negative results from using probiotics on allergic diseases are observed^31^, probiotic bacteria continue to represent the most promising intervention for primary prevention of allergic disease^32^. The present data implicate that probiotics *C. butyricum* may be involved in the improvement of the epithelial barrier function by regulating the expression of HDAC1 and Trek1 in the epithelial cells, based on a mechanism that this bacterium produce butyrate to inhibit the activities of HDAC1^33^. The data shed a new light on the studies of probiotics and epithelial barrier since the epithelial barrier function is a critical factor in maintaining the homeostasis in the body.

The biological mechanism in the modulation of epithelial barrier function by Trek1 is to be further investigated. The role of Trek-1 in airway and mucosal Na^+^ and Cl^−^ transport has been documented^34^. The mucosal Na^+^ and Cl^−^ transport is one of the major parameters to mirror the epithelial barrier functions, which is mainly reflected by the short circuit current^35^. The present data show that the levels of Trek1 in the nasal epithelia correlate with the changes of nasal epithelial barrier functions including the alteration of the short circuit current and the permeability to macromolecular proteins. Our data are in line with previous reports that HDAC1 can suppress Trek1^15^ and further supported by the present data that HDAC1 suppresses the expression of Trek1 in the nasal epithelia. The data are strengthened by the evidence that blocking HDAC1 attenuates the inhibitory effect of the IL-4-induced Trek1 suppression in the nasal epithelia.

Different results are reported, such as Schwingshackl et al indicate that exposure to hyperoxia accentuates lung injury in Trek1-deficient mice, but not controls. However, inverse results were also observed by Schwingshackl et al that combination of hyperoxia and injurious mechanical ventilation resulted in further morphological lung damage in controls but not Trek1-deficient mice^36^. The fact implicates that the role of Trek1 in modulating the epithelial barrier functions is dependent on the nature of stimuli; further studies are needed to elucidate the underlying mechanisms.

It is also reported that that Trek-1 deficiency has been shown to cause significant alterations in inflammatory cytokine secretion (IL-6, MCP-1, RANTES) from lung epithelial cells^36,37^. Although we utilized a different disease model in the present study, comparing with Schwingshacle's work, we have revealed another aspect of Trek1's properties that the expression of Trek1 can be suppressed by Th2 cytokines in the nasal epithelia. Whether such a suppression of Trek1 can further increase the levels of IL-6, MCP-1 and RANTES in the nasal epithelia as reported by Schwingshacle et al is worth being further investigated.

In summary, the present data indicate that nasal epithelia express Trek1; the latter plays an important role in the maintenance of the epithelial barrier integrity. Allergic mediator IL-4 can suppress the expression of Trek1 via increasing the expression of HDAC1 in the nasal epithelia. Administration of probiotic bacteria, *C. butyricum* prevents the allergic mediator IL-4-induced epithelial barrier dysfunction.

## Methods

### Reagents

The antibodies of Trek1 (C-20) and HDAC1 (C-19) were purchased from Santa Cruz Biotech (Shanghai, China). *Clostridium butyricum* (#1987252:285–289) was a gift from Shenzhen Kexing Biotech (Shenzhen, China). The recombinant proteins of IL-4 and HDAC1 were purchased from Biomart (Shenzhen, China). Sodium butyrate and horseradish peroxidase (HRP) was purchased from Sigma Aldrich (Shanghai, China). The ELISA kit of OVA-specific IgE was purchased from AbD Sertec (Shenzhen, China). The ELISA kits of IL-4, IL-5 and IL-13 were purchased from R&D Systems (Shanghai, China). The reagents for real time RT-PCR and Western blotting were purchased from Invitrogen (Shenzhen, China).

### Experimental animals

Sprague Dawley rats (200–250 g) were purchased from the Guangdong Experimental Animal Center. The rats were maintained in a pathogen-free environment. The experimental procedures were approved by the Animal Ethic Committee at Shenzhen University. The methods were carried out in accordance with the approved guidelines.

### Nasal allergy rat model

Rats were treated with ovalbumin (OVA; 5 mg/kg body weight in 1 ml alum) via subcutaneous injection at the back skin on day 0 and day 7 respectively. From day 14 to day 20, the rats were treated with a nasal drop containing OVA (1 mg/ml) at 0.1 ml/rat daily. Control rats were treated with saline in the same procedures above. The rats were sacrificed on day 21 and processed for further experiments as described below.

### Collection of nasal epithelial specimens

Following published procedures^38^, the nasal epithelial specimens were gently scraped from the surface of the inferior turbinate of 30 patients with allergic rhinitis (AR, diagnosed by their physicians; male 15; female 15) and 30 healthy subjects (male 15, female 15). Patients using anti-allergy agents, including steroids and anti-histamine, leukotriene receptor antagonists, etc. In the recent 2 months were excluded from the present study. In the same procedures, the epithelial specimens were also collected from the surface of the nasal septum of rats upon sacrifice. The using nasal tissue in the present study was approved by the Human Ethic Committee and Animal Ethic Committee at Shenzhen University. An informed, written consent was obtained from each human subject.

### Real time RT-PCR (qRT-PCR)

The total RNA was extracted from cells or nasal scrapings with the TRIzol reagents. The cDNA was synthesized with a reverse transcription kit. The qPCR was performed on a MiniOpticon qPCR device (Bio-Rad, Shanghai, China) with the SYBR Green Super Mix. The results were calculated with the 2^−ΔΔCt^ method and normalized to a percentage of the internal control β-actin. The primers using in the present study include: Human Trek1: forward, caattcgacggagctggatg; reverse, cttctgtgcgtggtgagatg. Human β-actin: forward, cgcaaagacctgtatgccaa; reverse: cacacagagtacttgcgctc. Rat Trek1: forward, cgccgtcatattcaagcaca; reverse, cccacctcttccttcgtctt. Rat β-actin: forward, tcttccagccttccttcctg; reverse, cacacagagtacttgcgctc.

### Western blots

One milligram nasal epithelial specimen was lysed with 0.1 ml lysis buffer (YiLi Biotech, Shanghai, China). The protein was quantitated by the Bio-Rad protein assay method. The protein (50 μg/well) was fractioned by SDS-PAGE (sodium dodecyl sulfate polyacrylamide gel electrophoresis; 12%) and transferred onto a PDVF membrane. After blocking with 5% skim milk, the membrane was incubated with the primary antibodies (100 ng/ml for HADC1 and 500 ng/ml for Trek1) for 1 h at room temperature and followed by incubation with the secondary antibodies (conjugated with horseradish peroxidase) for 1 h. The membrane was washed with TBST (Tris-buffered saline-Tween 20) after each incubation. The membrane was blotted with the enhanced chemilumescent (ECL). The results were recorded by the KODAK Image Station 4000 mm Pro. The integrated density of the immune blots was determined by the software of PhotoShop (CS5).

### Enzyme-linked immunosorbent assay (ELISA)

Cytokine levels were determined by ELISA with commercial reagent kits following the manufacturer's instructions.

### Assessment of rat nasal epithelial barrier functions

The short circuit current (*Isc*) and permeability to horseradish peroxidase (HRP) of the rat nasal septum mucosa were assessed in a mini Ussing chamber system (aperture diameter, 2 mm and square area, 0.0314 cm^2^) with our established procedures.

### Immunohistochemistry

The rat nasal mucosa was excised immediately after sacrifice and processed for cryosections. The sections were fixed in cold acetone for 20 min and blocked with 1% bovine serum albumin for 30 min. The sections were incubated with the primary antibodies (Trek1, 1:300; HDAC1, 1:300), or isotype IgG, at 4°C overnight and followed by secondary antibodies (FITC-labeled anti-goat IgG; 1:300) for 1 h at room temperature. Washing with phosphate buffered saline (PBS) (5 min, 3 times) was performed after each incubation. The sections were then stained with propidium iodide (PI; 5 μg/ml) for 5 min. After washing with PBS, the sections were mounted with cover slips and observed with a confocal microscope (LSM 510, Carl Zeiss). The slides were coded. The observers were not aware of the code to avoid the observer bias.

### Statistics

The data are presented as mean ± SD. Differences between groups were determined by one-way ANOVA. A p < 0.05 was set as a significant criterion.

## Electronic supplementary material

Supplementary Information
supplemental materials
