# Supplementary material for: Trek1 contributes to maintaining nasal epithelial barrier integrity
Source: Sci Rep. 2015 Mar 17;5:9191. doi: 10.1038/srep09191 (PMC7365316; doi:10.1038/srep09191)
Supplement: Supplementary file 1 — Supplementary Information supplemental materials [file 41598_2015_BFsrep09191_MOESM1_ESM.pdf]

Supplemental materials

Trek1 contributes to maintaining nasal epithelial barrier integrity

Running title: Trek1 and nasal epithelial barrier function

Jing Jiang *[abc], Jiang-Qi Liu *[abc], Jing Li *[a], Meng Li [a], Hong-Bin Chen [a], Hao Yan [a], Li-Hua Mo [a], Shu-Qi Qiu [ab], Zhi-Gang Liu [a], Ping-Chang Yang [ac]

[a]ENT Institute of Shenzhen University and Shenzhen Key Laboratory of Allergy & Immunology, Shenzhen, 518060, China. [b]ENT Hospital, Longgang Central Hospital, Shenzhen, 518116, China. [c]Brain Body Institute, McMaster University, Hamilton, ON, Canada L8N 4A6.

*These authors contributed equally to this work.

Corresponding authors: Dr. Ping-Chang Yang and Dr. Zhi-Gang Liu (Room 204 of Medical School Building; 3688 Nanhai Ave., Shenzhen, 518060, China. Email: pcy2356@szu.edu.cn. Tel: 86 755 8617 2722. Fax: 86 755 8617 1906).

Full length gels (were photographed by the KODAK Image Station 4000 mm Pro).

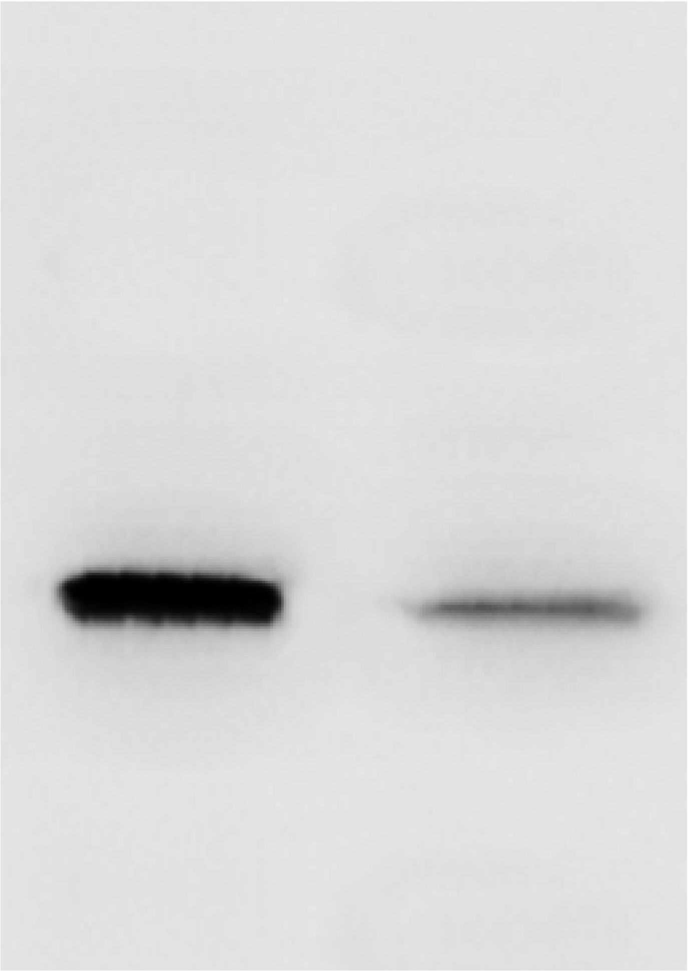

Fig. 1B-Trek1

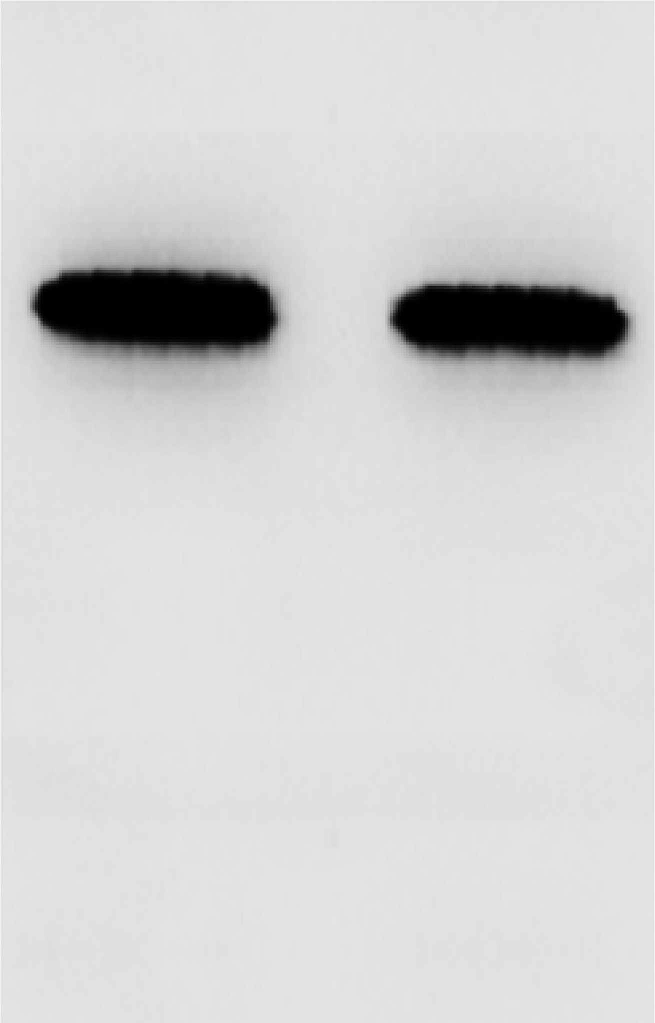

Fig. 1B-Beta actin

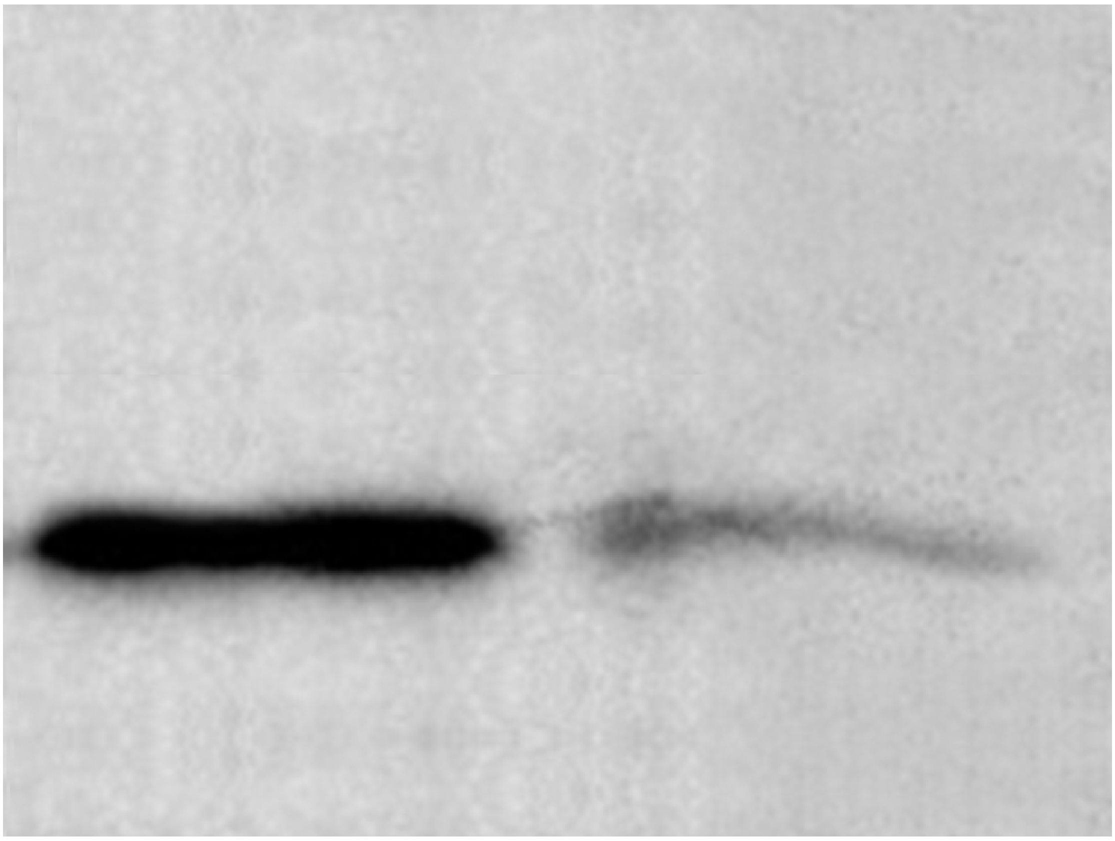

Fig. 2C-Trek1

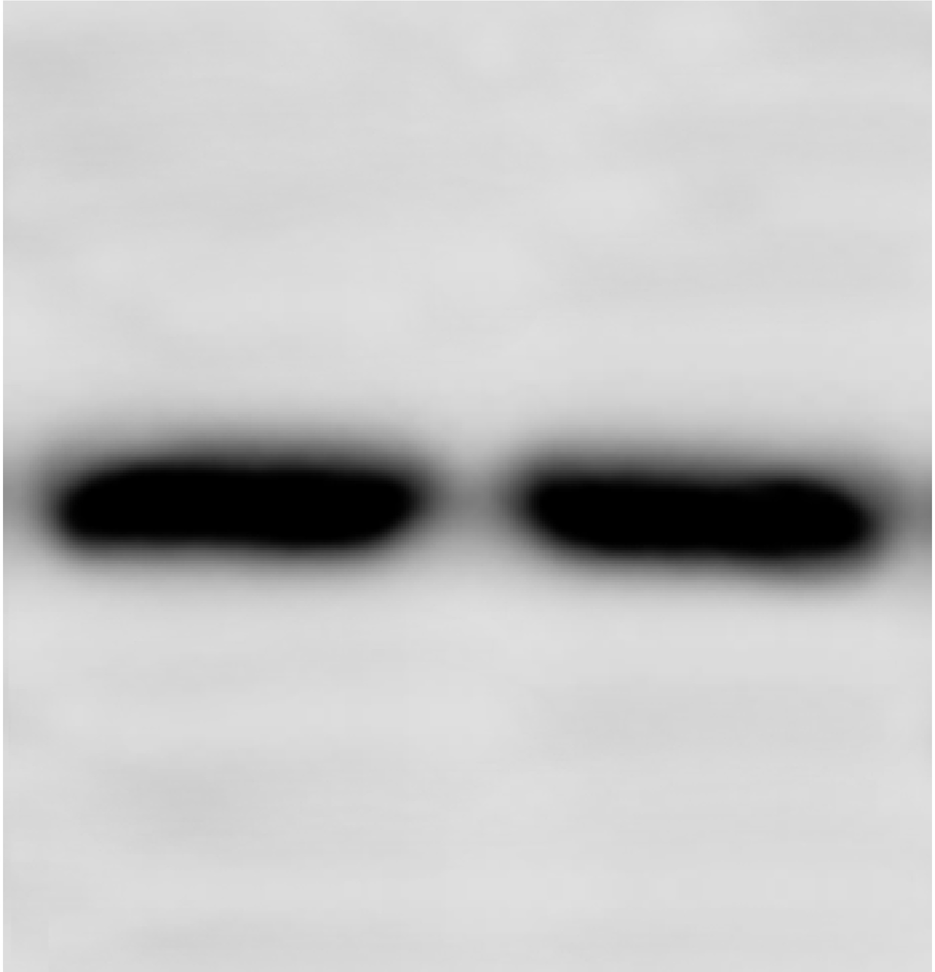

Fig. 2C-beta actin

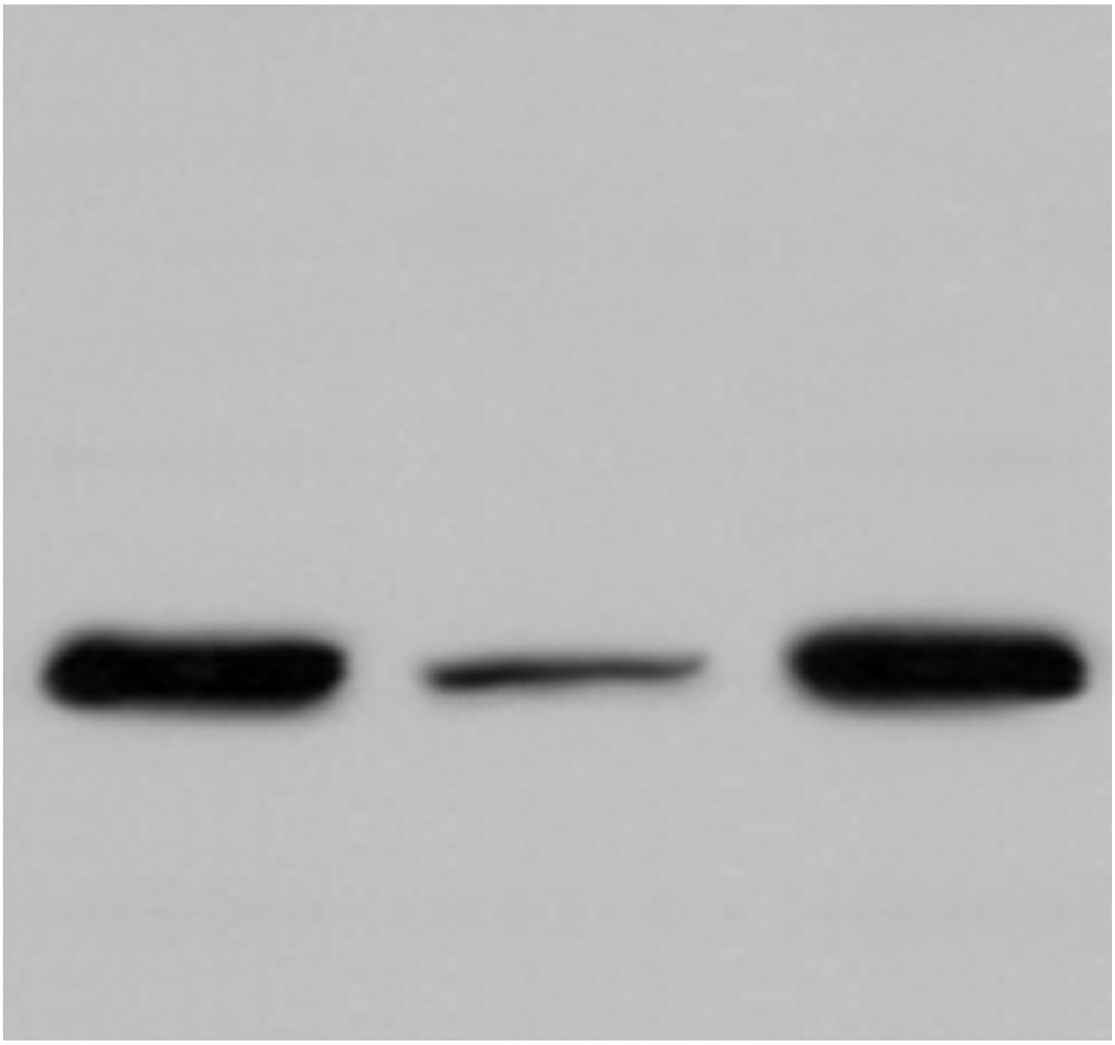
Fig. 3B-Trek1

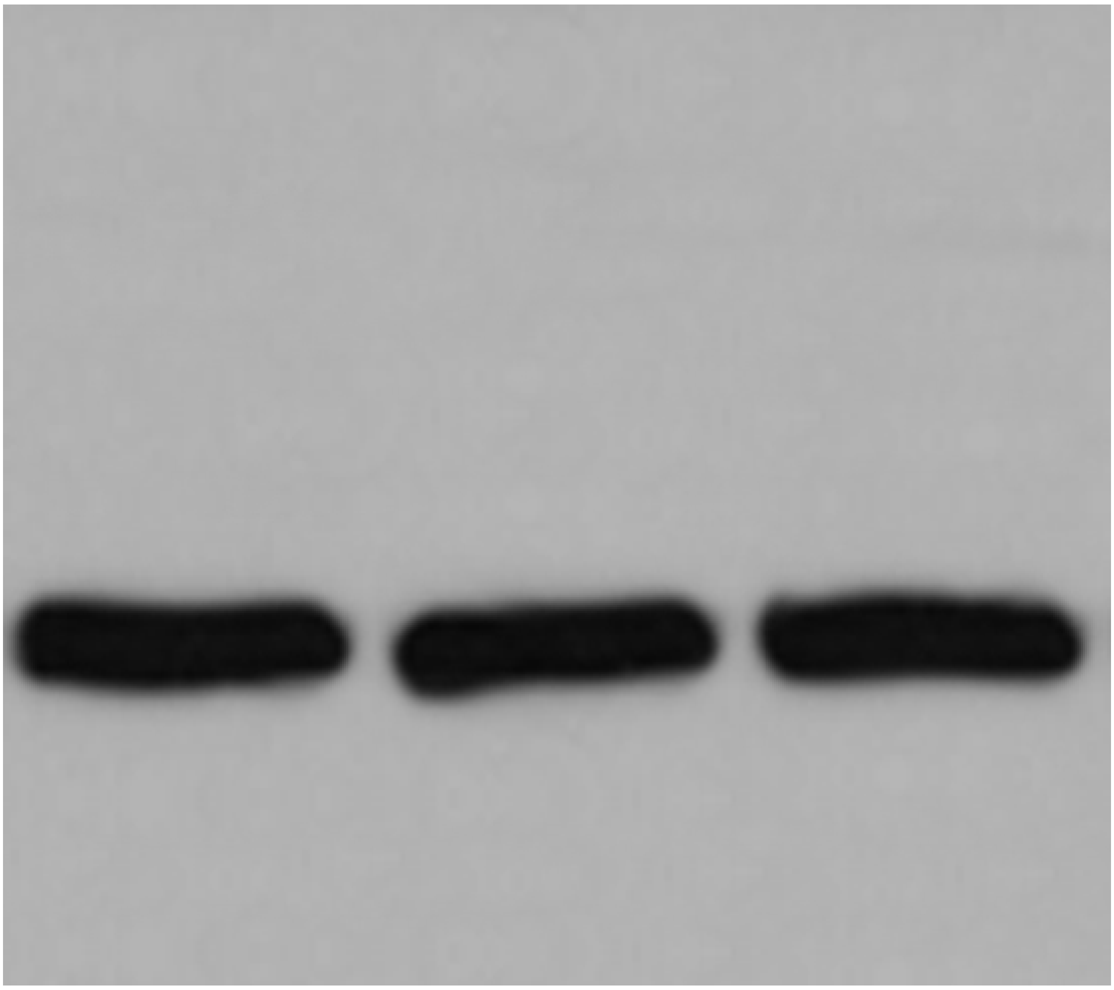

Fig. 3B-beta actin

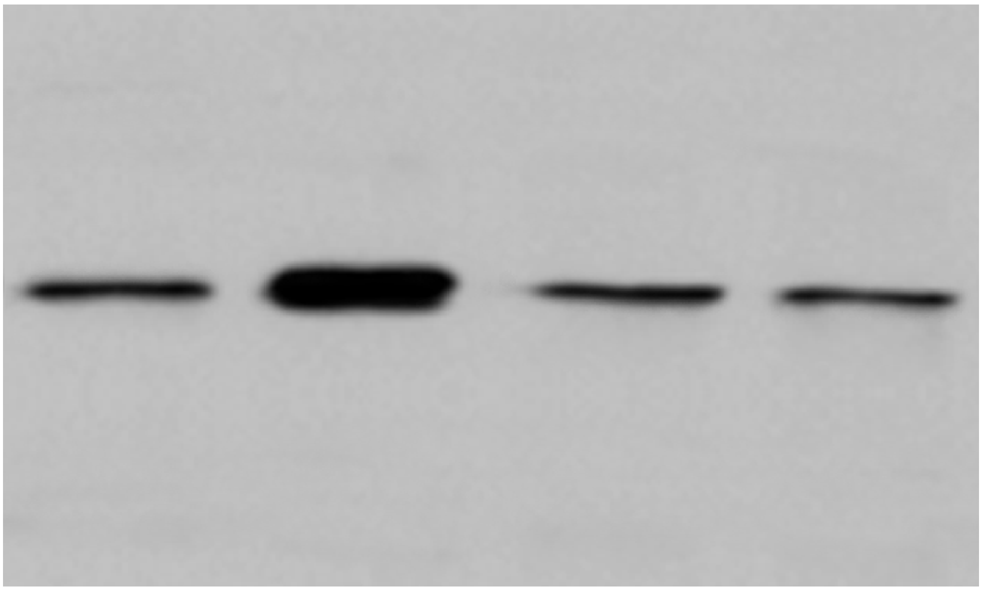

Fig. 3C-HDAC1

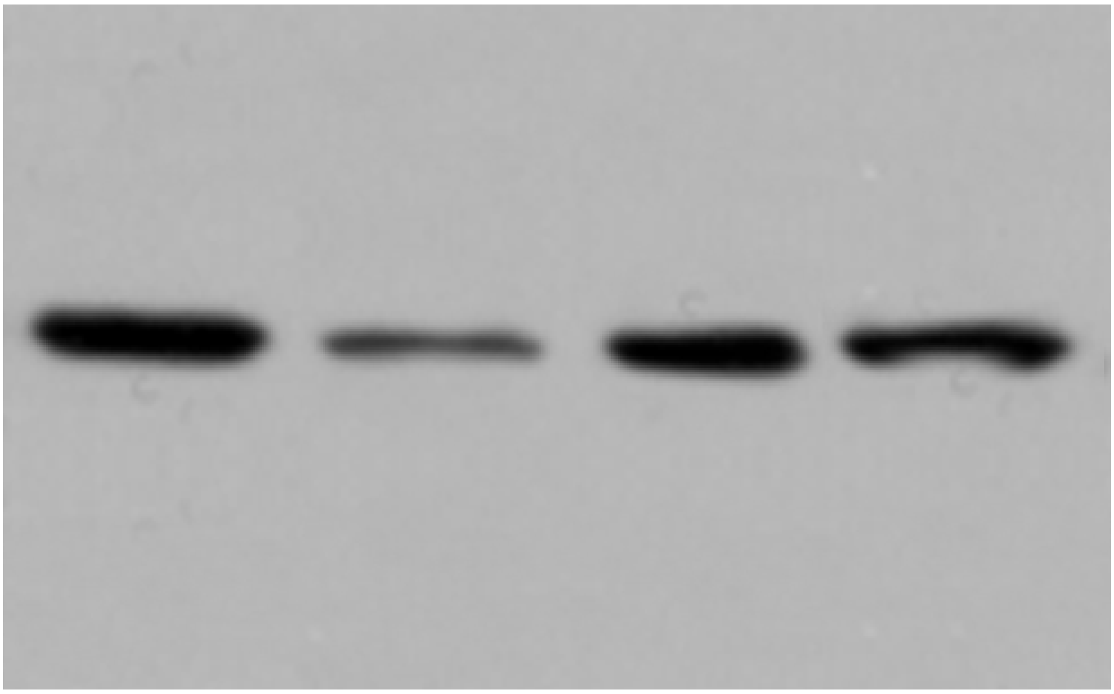

Fig. 3C-Trek1

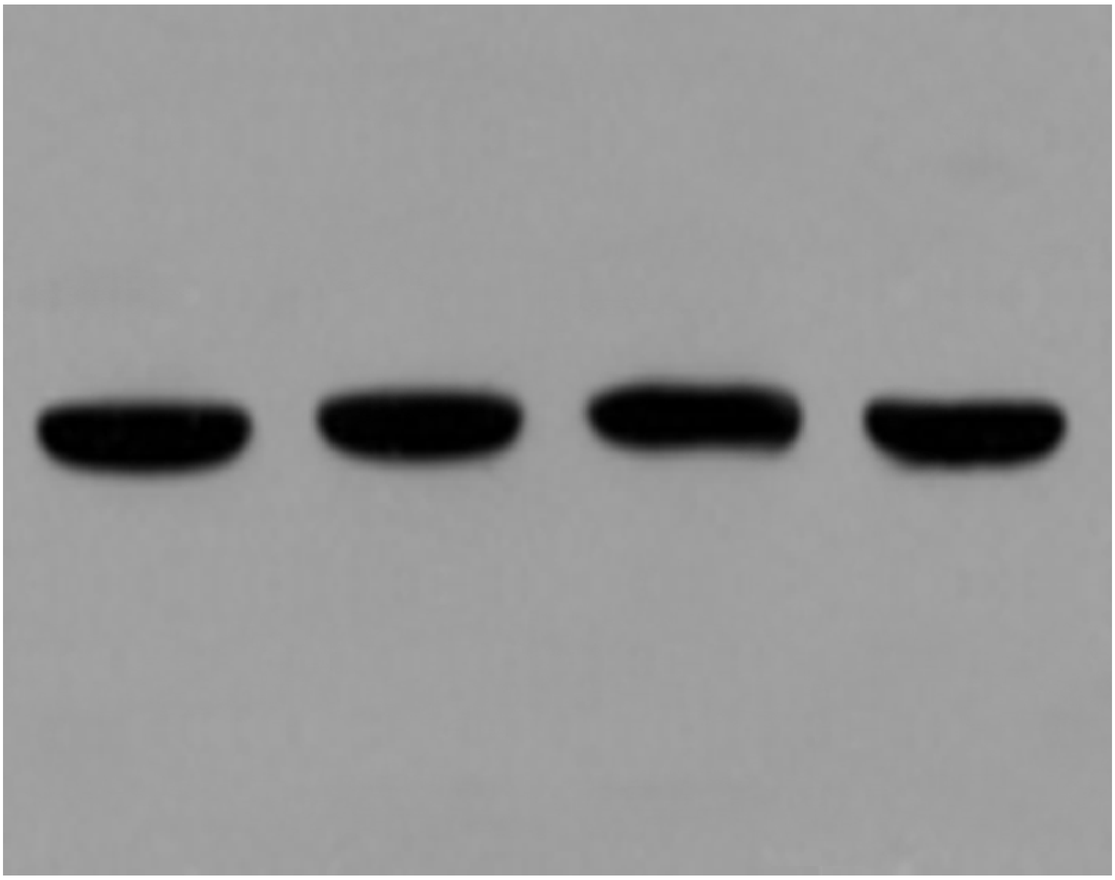

Fig. 3C-beta actin
